# What goes around comes around: updates in the study of *Coxiella burnetii* lipopolysaccharide phase variation

**DOI:** 10.1128/iai.00030-26

**Published:** 2026-08-19

**Authors:** Kathleen N. Pierce, Rebecca Anderson, Megan Brose, Jessica McCormick-Ell, Carrie Mae Long

**Affiliations:** 1 Laboratory of Bacteriology, Division of Intramural Research, National Institute of Allergy and Infectious Disease, National Institutes of Health 469049, Hamilton, Montana, USA; 2 Department of Microbiology and Immunology, University of Arkansas for Medical Sciences 318223, Little Rock, Arkansas, USA; 3 Division of Safety, Office of Research Services, National Institutes of Health 116041, Hamilton, Montana, USA; 4 Division of Safety, Office of Research Services, National Institutes of Health 116041, Bethesda, Maryland, USA; University of California at Santa Cruz, Santa Cruz, California, USA

**Keywords:** *Coxiella*, Q fever, LPS, biosafety, vaccines, gram-negative bacteria, pathogenesis, lipopolysaccharide, select agent, BSL-3

## Abstract

Lipopolysaccharide (LPS) is a major outer membrane component of Gram-negative bacteria that can modulate host immune responses during infection. Extensive studies on the LPS of well-studied organisms, such as *Escherichia coli* and *Salmonella* spp., have elucidated many LPS pathways; however, knowledge of unique LPS structures of lesser-studied bacteria, such as *Coxiella burnetii*, remains limited. *C. burnetii* is an intracellular pathogen that is the cause of the zoonotic disease Q fever. The highly infectious nature of *C. burnetii* warrants designation as a select agent by the United States Federal Select Agent Program and must be contained in a Biosafety Level three facility. Contributing to the organism’s biothreat potential, *C. burnetii* LPS is a critical virulence determinant that dictates bacterial survival and pathogenesis. Several reviews have extensively covered *C. burnetii* LPS structure and function prior to 2017, though recent findings regarding mechanisms of LPS biosynthesis, LPS-induced host immunity, and LPS elongation pose biosafety and regulatory questions that necessitate careful consideration. Here, we discuss recent (post-2017) findings related to *C. burnetii* LPS from scientific and biosafety perspectives that may be applicable across many fields of research. From the discovery of *C. burnetii* LPS phase variation by Fiset and Stoker in 1956 to the recent elucidation of specific genetic mechanisms underlying this process, the study of *C. burnetii* LPS is a reminder that what goes around comes around, even in research.

## INTRODUCTION

Lipopolysaccharide (LPS) is a major outer membrane component of Gram-negative bacteria with functional importance in the context of human disease. Indeed, the biological effects of “endotoxin” were described in the late 1800s (1). Endotoxin, an early moniker for LPS, consists of four distinct domains: (i) lipid A, (ii) inner core oligosaccharide, (iii) outer core oligosaccharide, and (iv) O-antigen (2). Lipid A confers unique outer membrane structural integrity and serves as a permeability barrier, thereby contributing to bacterial survival and propagation (3). Furthermore, LPS can modulate host immune responses in both stimulatory and suppressive manners, facilitating pathogen success during infection. The major determinant of LPS immunogenicity is lipid A. LPS is a pathogen-associated molecular pattern (PAMP) that can activate several innate immune pathways, including toll-like receptor 4 (TLR4) signaling, caspase cleavage, and complement system activation (4–7). This stimulatory effect on the innate immune response is particularly beneficial for fighting infections and, when used as an adjuvant, can augment adaptive immune responses to vaccination (8, 9). These pathways also rapidly amplify one another, inducing sepsis and leading to multiple organ failure via endotoxic shock (10), demonstrating the potent immunostimulatory potential of this molecule. *Escherichia coli* and *Salmonella* spp. are two model organisms for the study of LPS, and related studies have identified specific extracellular and cell membrane mediators of LPS-activated proinflammatory pathways, such as serum LPS-binding protein (LBP), CD14, and the MD2/TLR4 complex (11–14). Intracellularly, caspases 4 and 5 in humans and caspase 11 in mice are responsible for pyroptosis and NLRP3 activation, both of which amplify inflammation independent of TLR signaling (15–18). LPS structural studies have focused on these well-studied organisms, and while many bacteria express LPS similar structures, variations can occur via lipid A modification, sugar variation, and differential expression of O-antigen polysaccharides (also known as phase variation). For example, phase variation is often described as a smooth-to-rough transition, meaning that LPS with full-length O-antigen becomes truncated, generally leading to bacterial attenuation. Phase variation typically occurs through repeated passaging in conditions that lack selective pressures, such as those exerted by the host immune system in intact organisms. While many Gram-negative bacteria can undergo phase variation, both bacterial and host determinants of this process vary among species.

Despite seminal work on LPS in model organisms, many questions remain regarding the unique LPS structure and function of bacteria such as *Coxiella burnetii*, an intracellular bacterium that causes the zoonosis Q fever. The disease is characterized by a high fever and flu-like symptoms, though most acute cases are asymptomatic and remain underdiagnosed (19, 20). Chronic Q fever, though less common, is characterized by persistent focalized infections that can cause life-threatening complications (21). *C. burnetii* is an obligate intracellular bacterium that spreads through aerosol transmission and has a human infectious dose below 10 organisms (19). Due to high environmental stability and infectivity, *C. burnetii* is considered a potential biological warfare agent and is regulated by the United States Federal Select Agent Program (FSAP) (22–24). Virulence factors of *C. burnetii* that mediate host-pathogen interactions include LPS and the secretion of effector proteins into the host cytoplasm by a Type 4B Secretion System (T4BSS) (25–27). Indeed, *C. burnetii* LPS length is a major determinant of bacterial virulence, as elongated LPS allows for immune evasion during infection (25, 28, 29). *C. burnetii* LPS is primarily categorized into three phases: full-length, virulent phase I LPS (PhI), intermediate-length, mildly attenuated intermediate phase LPS (PhInt), and truncated, attenuated phase II LPS (PhII). *C. burnetii* LPS also undergoes typical (smooth-to-rough) phase variation, first described as antigenic variation in serum responsiveness (30); however, studies on the mechanisms underlying LPS biosynthesis and phase variation are ongoing and major knowledge gaps remain.

In this review, we will discuss selected research milestones, recent developments, and remaining questions regarding *C. burnetii* LPS. From structure to function, the general study of LPS offers powerful insights into both bacterial and human adaptation, disease, and survival. The study of *C. burnetii* LPS is no different; thus, we provide insight into recent developments pertinent to *C. burnetii* LPS structure and function as related to host-pathogen interactions and countermeasure development. This review focuses on developments since 2017, as earlier developments have been comprehensively described elsewhere (31, 32). We contextualize these findings in relation to emerging biosafety and scientific questions with cross-disciplinary implications, providing commentary regarding the future study of *C. burnetii* LPS.

## A BRIEF PRIMER ON HISTORIC MILESTONES IN THE STUDY OF *C. BURNETII* LPS

Concordant with the identification of the causative agent of Q fever, *C. burnetii*, in 1937 by research groups in Australia and the United States, basic characterization revealed the Gram-negative nature of the bacterium (33, 34). The organism’s current name, *Coxiella burnetii*, was not proposed until 1948 (35); thus, former names include the Nine Mile agent, the Q fever virus, *Rickettsia diaporica*, and *Rickettsia burneti* (36). Importantly, *C. burnetii* was likely the “filter-passing virus” derived from *Dermacentor andersoni* ticks identified by Dr. Hideyo Noguchi in 1926 in collaboration with scientists at the Rocky Mountain Laboratories in Montana, USA (37). Due to a lack of suitable growth media, detailed characterization of this organism was not feasible; however, Gram-negative organisms were observed in infectious suspensions prior to attempted egg passage. Davis and Cox reported the Gram-negative, *Rickettsia*-like nature of *C. burnetii* in basic bacteriological testing (38, 39).

Nearly two decades following *C. burnetii*’s discovery, serologic phase variation was characterized by Fiset and Stoker (30). This phenomenon was described as altered responsiveness to the serum complement fixation assay, correlated with repeated egg yolk passaging of *C. burnetii*. Here, PhI and PhII organisms were defined based on responsiveness, with PhI isolates recovered from *in vivo* infections and PhII isolates appearing after repeated egg culture. A year later, Fiset reported further characterization of phase variation, hypothesizing that this phenomenon was associated with loss of surface antigen (40). This prescient observation foreshadowed the correlation of LPS structure with serologic phase variation. Functional variation in PhI and PhII isolates was observed by Kazar et al., in the context of enhanced phagocytosis by macrophages and polymorphonuclear leukocytes (41). In 1982, cell envelope and LPS sugar compositional analysis provided insight into the relationship between LPS phase variation and LPS structure (42, 43). This analysis followed that of Baca et al., providing a more detailed compositional portrait of LPS phases (44). The *C. burnetii* O-antigen sugars virenose and dihydroxystreptose were identified in 1985 and have not been described in other bacteria, demonstrating the uniqueness of LPS from this organism (45). In the same year, *C. burnetii* LPS fatty acids were analyzed, revealing the unique fatty acid spectrum of *C. burnetii* among Gram-negative bacteria (46). *C. burnetii* O-antigen can differ in size and composition between PhI isolates, compared with many bacteria that have repeating O-antigens (47–49). Given this variability, the exact structure of the *C. burnetii* O-antigen remains poorly characterized. Building on observations by Schramek and Meyer, Toman et al. identified KDO (3-deoxy-D-manno-2-octulosonic acid) in PhII LPS (43, 50). Zamboni et al. demonstrated that PhII *C. burnetii* activates toll-like receptor 2 (TLR2) but not TLR4 in Chinese hamster ovary/CD14 cells, revealing resultant cytokine production upon TLR2 stimulation (51). Importantly, this study also revealed the homogeneity of lipid A from PhI and PhII variants via electrospray ionization-mass spectrometry analysis and the lack of TLR2 or TLR4 stimulation by lipid A from both phase variants *in vitro*. A 2009 study by Vadovik et al. described the lipid A structure from Nine Mile Crazy (NMC), a *C. burnetii* strain expressing PhInt LPS that is genetically similar to the Nine Mile I (NMI, expressing PhI LPS) reference strain apart from virenose (52). NMC lipid A was compositionally similar to that of NMI and Nine Mile II (NMII, expressing PhII LPS); however, the authors note that the lipid A proximal region of NMC LPS is more chemically complex than that of PhII LPS. The proposed lipid A structure of *C. burnetii* is a glucosamine disaccharide backbone, similar to *E. coli. C. burnetii* lipid A is tetra-acylated and is considered a “poor” endotoxin, in contrast to hexa-acylated lipid A structures (e.g., *Klebsiella pneumoniae*, *Salmonella* spp., and *E. coli*), which induce strong pro-inflammatory responses (53–55). Adding to the reduced endotoxicity demonstrated by *C. burnetii* lipid A (56), various immunoevasive mechanisms are associated with PhI LPS. A seminal study by Moos and Hackstadt solidified PhI LPS as a critical *C. burnetii* virulence determinant *in vivo* (25). As a virulence determinant, PhI *C. burnetii* LPS can sterically inhibit antibody binding to the surface *C. burnetii* (28) and can act as an antigen shield in interactions with dendritic cells (29). Altogether, LPS length is a critical virulence determinant, with longer length corresponding with enhanced virulence.

Since its identification, *C. burnetii* phase variation has been thoroughly studied in the context of protective immunity and vaccine development. Ormsbee et al. demonstrated the disparity in whole cell vaccine (WCV) potency dependent on LPS phase, where PhI WCVs demonstrated 100–300 times greater potency than their PhII WCV counterparts (57). Over two decades later, *C. burnetii* LPS structure and antigenicity were shown to be heterogeneous among genetically distinct bacterial isolates (47, 58); however, protective immunity was retained among all PhI strains, suggesting that conserved immunogenic epitopes are present. In 2013, Zhang et al. characterized immunologic mechanisms of PhI WCV (subcutaneous delivery) in mice, revealing that both T cell-independent and T cell-dependent mechanisms were required for protection following intraperitoneal challenge (59). The importance of PhI LPS in vaccine efficacy remains a critical concept in Q fever vaccine design. While PhI WCVs remain the gold standard for protection, LPS-targeted vaccine designs have been explored, including LPS peptide mimics (60), adjuvanted soluble antigen extracts (61), purified LPS (62), and adjuvanted LPS glycoconjugate vaccines (63). Importantly, the PhI antigen appears to be critical for maximal vaccine-induced protection and durability.

**Fig 1:**
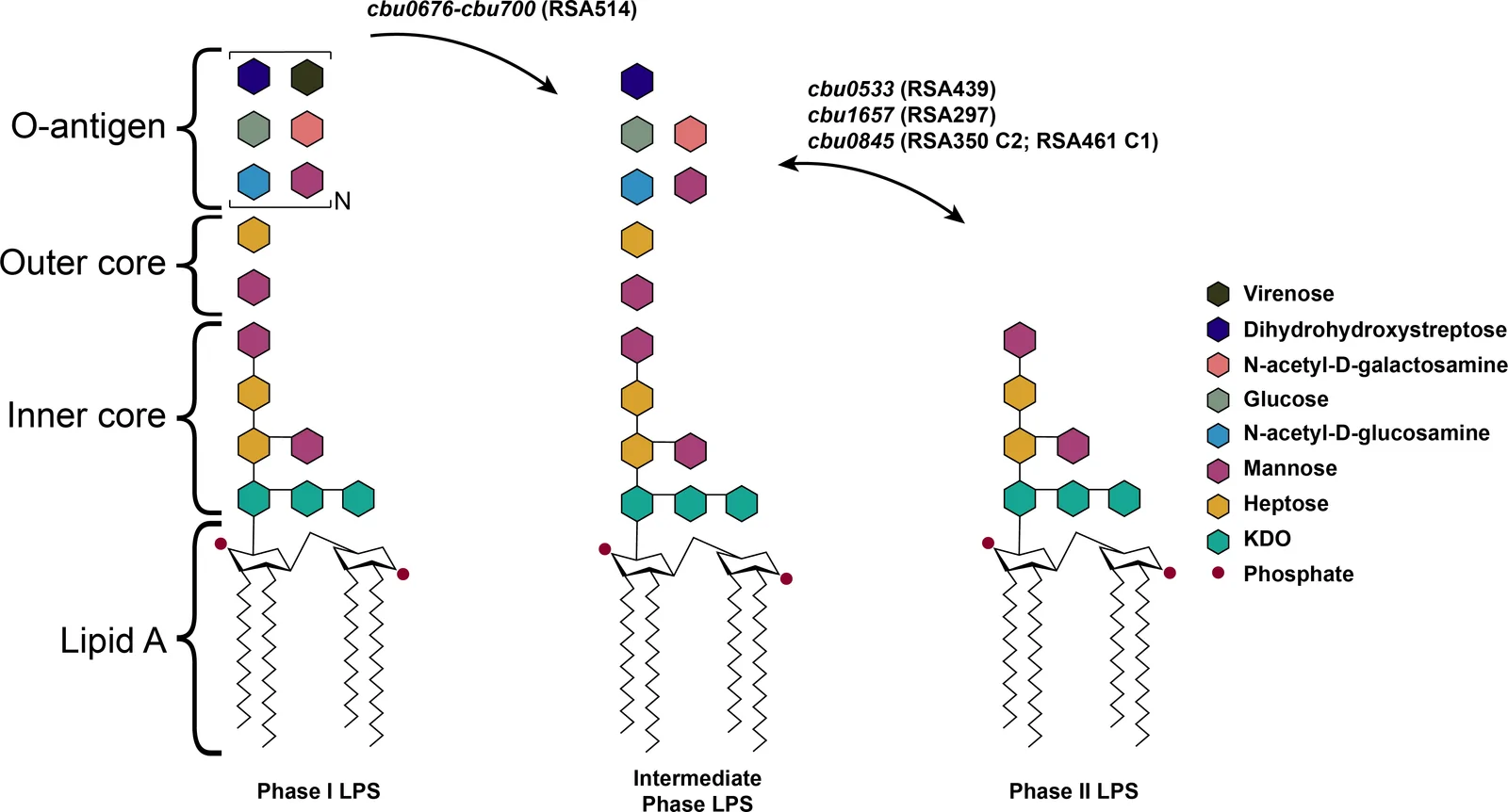
Genetic mechanisms governing *C. burnetii* LPS phase variation, with respective isolates denoted.

Concordant with the molecular biology revolution during the turn of the 21st century, Hoover et al. described a large chromosomal deletion found in the NMII and NMC strains (64). This ~38 kb deletion spanned LPS biosynthesis genes, revealing a component of the genetic basis of LPS truncation, but not fully explaining the transition to PhII LPS (65). In 1984, a clonal variant of the PhII strain, NMII, was introduced (66). This NMII, clone 4 strain was thought to be avirulent due to its truncated LPS profile, a hypothesis supported by Moos and Hackstadt in guinea pig infection studies in which NMII intraperitoneally inoculated animals did not show signs of disease and viable bacteria were not isolated from guinea pig spleens (25). These attributes of NMII, clone 4 led to select agent status exemption from United States Centers for Disease Control and Prevention/United States Department of Agriculture (US CDC/USDA) FSAP restrictions in 2003 (67). This decision enabled biosafety professionals to permit the use of this strain in biosafety level 2 (BSL-2) laboratories, based on risk assessment and in accordance with other United States Government (USG) requirements. Further investigation of the genetic determinants of *C. burnetii* LPS phase variation revealed minor genetic alterations responsible for phase variation in PhII Australian strains (68), prompting further study of the genetic mechanisms of this phenomenon. A visual summary of selected milestones in *C. burnetii* LPS research is depicted in Fig. 1. This list is not exhaustive and was formed based on the authors’ perspectives.

## RECENT DEVELOPMENTS IN THE STUDY OF *C. BURNETII* LPS

Currently, much remains unknown regarding *C. burnetii* LPS structure, function, and phase variation. Clearly, this molecule is critical to many aspects of *C. burnetii* biology and host responses, justifying further study. Next, we will describe recent (post-2017) developments in *C. burnetii* research related to the understanding of LPS.

### LPS genetics

Several studies in the early 2000s focused on characterizing LPS structural variations of different isolates and their related bioactivity, along with initial genetic characterization of *C. burnetii* strains in the context of LPS phase (29, 52, 64, 68–73); however, we will provide an in-depth focus on post-2017 findings that supported previous hypotheses suggested regarding LPS biosynthesis and phase variation (49, 74).

**Fig 2:**
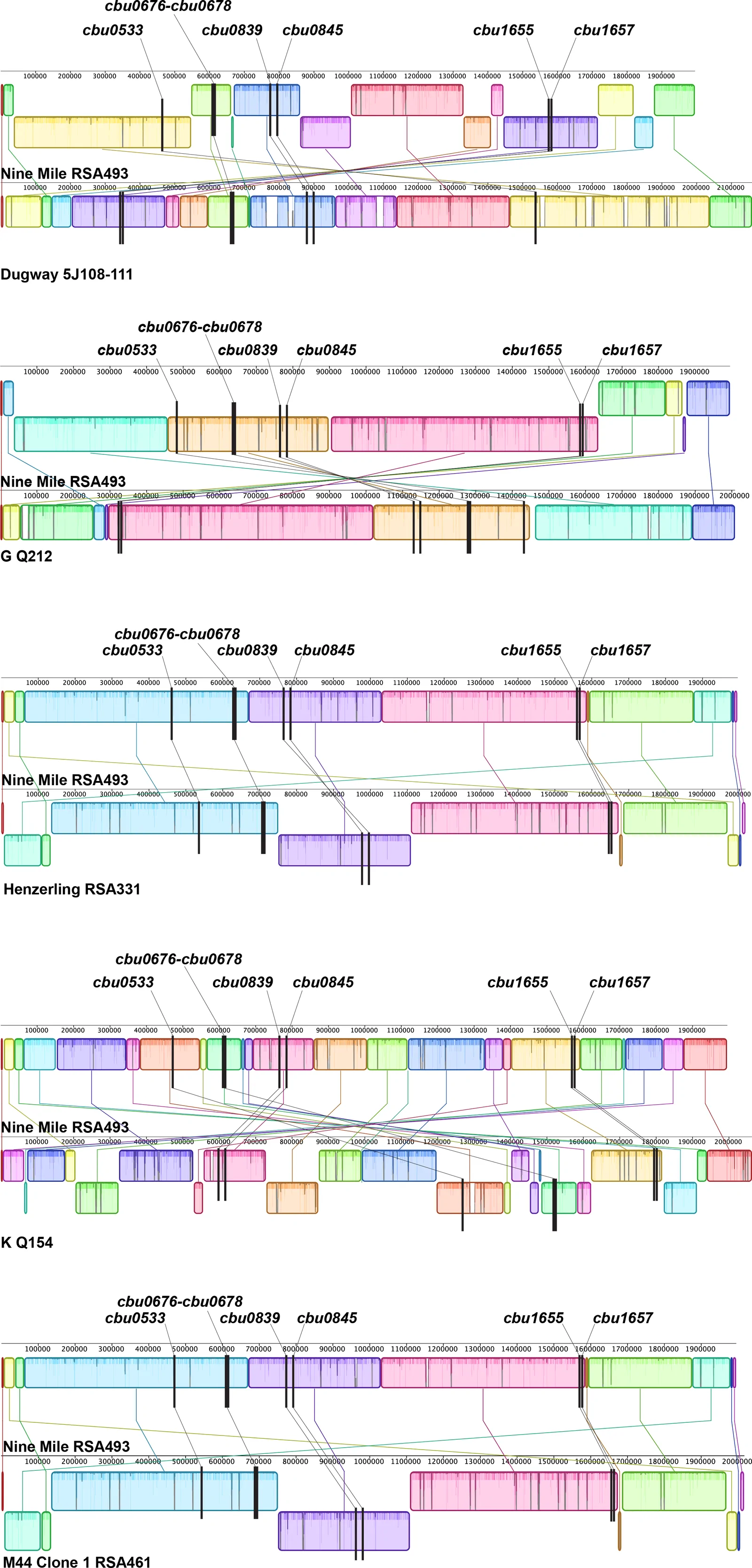
Locations of genes responsible for LPS phase variation in various *C. burnetii* isolates compared with the Nine Mile I RSA493 strain.

Vodkin and Hoover previously reported that large chromosomal deletions in NMII and NMC were related to LPS phase variation, though the lack of large chromosomal deletions in some strains with PhII reactivity led them to suggest that point mutations or mutations outside these deletions could also be responsible for PhII LPS (64, 65, 75). This was supported by whole-genome sequencing, revealing the lack of a large deletion in Australia QD RSA425 clone three despite PhII reactivity, suggesting that a specific SNP or SNPs were responsible for LPS phase transition (64, 68). In 2018, Beare et al. defined several mutations responsible for LPS phase transition through mutational analyses across several *C. burnetii* genomic groups (Fig. 1) (49). Whole-genome sequencing revealed mutations related to truncated LPS production (Table 1). A novel nutritional genetic selection system was utilized to demonstrate the functional significance of these mutations for LPS biosynthesis. This analysis also revealed mutations in *cbu0678* in isolates expressing PhI and PhInt LPS, and targeted mutagenesis established *cbu0678* as essential for PhI LPS expression and virenose production. It is also notable that this gene is thought to be acquired by horizontal gene transfer from members of Alphaproteobacteria, based on maximum likelihood and Bayesian phylogenetic analyses from Moses et al., and was identified as important for *C. burnetii* infection in a *Galleria mellonella* hemocyte infection model (Table 1) (76, 77). Mutations in *cbu1657* were observed in Australian strains; this gene is suspected to encode an alpha-L-*glycero*-D-*manno*-heptose beta-1,4-glucosyltransferase, which transfers glucose to heptose I in the inner core in *K. pneumoniae* (78). PhII LPS-related mutations were also seen in *cbu0845*, which is suggested to be required for mannose production. Complementation of *cbu0845* resulted in production of PhInt LPS, further supporting the hypothesis that PhI transition to PhII is a multi-step process (49). Repeated passing of NMC RSA514 showed accumulation of a 3-bp deletion in *cbu0533*, resulting in PhII LPS. The accumulation of *cbu0533* mutations was also seen in PhII isolates Turkey RSA315, Ohio RSA338, and NMII RSA439 clone 4. This suggests that *cbu0533* is a common site for mutations resulting in phase variation. *Cbu0533* shares homology with the undecaprenyl-phosphate alpha-N-acetylglucosaminyl transferase *wecA* in *E. coli*, though it was reported that CBU0533 could not be successfully complemented into *E. coli* O157:H7 ZAP198Δ*wecA in trans* (31). The genes characterized in these studies and their location in the genomes of various *C. burnetii* isolates relative to *C. burnetii* Nine Mile I RSA493 are depicted in Fig. 2. Collectively, this work represents a major shift in the understanding of genetic mechanisms underlying *C. burnetii* LPS phase variation, enabling subsequent discoveries linking genetics to function.

**TABLE 1:** *C. burnetii* genes related to LPS biosynthesis (reported post-2017)

*Coxiella burnetii* gene	Suspected gene homology	LPS phenotype of mutant	Essential for PhI LPS	Publications
*cbu0533*	WecA, undecaprenyl-phosphate alpha-N-acetylglucosamine phosphotransferase	Ph2	Yes	(49, 74, 79, 80)
*cbu0664* to *cbu0704*	Synthesis of virenose			(49)
*cbu0676*	UDP-glucose 4-epimerase	Suggested Ph2	Suggested	(49, 76, 77)
*cbu0677*	NAD-dependent epimerase/dehydrogenase family	Suggested Ph2	Suggested	(49, 77)
*cbu0678*	D-glycero-D-manno-heptose-1-phosphate adenylyltransferase, implicated in virenose biosynthesis	Ph2/PhInt	Yes	(49, 76, 77)
*cbu0825* to *cbu0856*	Synthesis of dihydrohydroxystreptose			(49)
*cbu0839*	Glycosyltransferase	Ph2		(49)
*cbu0841*	Alpha-D-QuiNAc alpha-1,3-galactosyltransferase			(49)
*cbu0844*	UDP-N-acetylglucosamine 4-epimerase			(77)
*cbu0845*	GDP-mannose dehydrogenase family, mannose synthesis	Ph2/PhInt		(49)
*cbu0846*	UDP-glucose-6-dehydrogenase			(77)
*cbu1655*	D-glycero-D-manno-heptose-7-phosphate 1-kinase/D-glycero-D-manno-heptose-1-phosphate adenylyltransferase, HldE	Ph2/R	Yes	(49, 77)
*cbu1657*	Alpha-L-glycero-D-manno-heptose beta-1,4-glucosyltransferase	Ph2/PhInt	Suggested	(49, 76)
*cbu1831* to *cbu1838*	Sugar components of O antigen			(49, 76)

In 2024, the phenomenon of *C. burnetii* LPS elongation was observed by silver stain and immunoblot analysis following guinea pig infection and during continuous axenic passaging (74). Whole-genome sequencing revealed that *cbu0533* can undergo a slipped-strand mutation reversion (insertion of 3 bp pairs, leucine at AA168) to wild-type *cbu0533* in NMII. This mutation reversion resulted in LPS elongation, like that of intermediate LPS-expressing strains, such as NMC RSA514, resulting in enhanced virulence *in vivo*. Specifically, LPS elongation due to *cbu0533* mutation reversion was observed following guinea pig infection and axenic passaging (ACCM-1 media). These findings were immediately reported to the National Institutes of Health (NIH) biosafety team and internal NIH committees for review, in addition to the FSAP, since some NMII strains with elongated LPS exhibited increased virulence compared with wild-type NMII. This resulted in a Federal Register Notice classifying any strain with the *cbu0533* mutation reversions as select agents and reinstating BSL-3 containment (81). As the NMII RSA439, clone 4 isolate is a commonly used laboratory workhorse strain, this ruling created potential logistical hurdles related to the manipulation of NMII RSA439 clone 4. Pierce et al. evaluated a NMII RSA439 clone 4 Δ*cbu0533* strain for infection phenotype *in vitro* and reported that this strain exhibits analogous growth and infection characteristics to wild-type NMII, RSA439 clone 4 (79). Further, Beare et al., reported the genome of this strain in 2025, confirming the complete deletion of *cbu0533* (80). Guinea pig infection studies suggest that the NMII, RSA439 clone 4 Δ*cbu0533* strain cannot undergo LPS elongation, making this strain an attractive alternative to wild-type NMII RSA439 clone 4, whose *cbu0533* mutation status is not fixed. Research involving this strain would enable safe BSL-2 work and eliminate concerns about mutation reversion, eliminating the need to follow select agent regulations. This strategy has been described by several groups studying *Mycobacterium tuberculosis*, who have created BSL-2-compliant strains of *M. tuberculosis* via genetic modification (82, 83). Indeed, Vilcheze et al. suggest that BSL-2-rated strains are “valuable to research laboratories” and could be useful for training in clinical laboratories (83).

### LPS and host signaling and immunity

Recent advancements in the study of *C. burnetii* LPS highlight functions beyond virulence, including impacts on host cell signaling and protective immunity. A 2018 study targeted NMII lipid A *in vitro* infection models via chemical inhibition of LpxC, an enzyme involved in lipid A biosynthesis (84). Further modulation of *C. burnetii* lipid A by *kdtA* from *Chlamydia* spp. resulted in anti-LpxC treatment-induced growth defects. This phenotype was only observed during prolonged anti-LpxC treatment and was reversible. The authors concluded that lipid A is important for *C. burnetii* vacuole formation in phagocytic cells.

Full-length *C. burnetii* LPS is a critical component of whole cell vaccine (WCV)-induced protective immunity. Post-2017 studies furthered understanding of this concept by elucidating related mechanisms. A 2019 study compared adaptive immune responses in mice immunized with PhI and PhII WCV (85). Microarray, ELISpot, and functional immune analyses were performed with sera and secondary lymphoid tissues. These analyses revealed 17 PhI WCV-specific, 6 PhII WCV-specific, and 267 shared humoral antigens. PhI WCV immunization resulted in an increased frequency of CD4 + IFN-γ- producing T cells, which are known to be involved in protective responses to *C. burnetii*. Like the results of Shannon et al. with human dendritic cells and live *C. burnetii* treatment (29), murine bone marrow-derived dendritic cells were differentially stimulated by PhI and PhII WCVs. Immunization of chemokine receptor 7 (CCR7) knockout mice revealed the loss of PhI WCV-induced adaptive immune potency, with impaired bacterial clearance following challenge. Although the route of immunization was not addressed, these studies suggest a general role for CCR7 in PhI WCV-induced immunity and support the hypothesis that LPS-related adaptive immunity is critical for WCV effectivity. Additional murine LPS-related vaccine efforts include the demonstration of live NMII as a vaccine candidate (86). After immunizing mice with live NMII via intraperitoneal, intranasal, intramuscular, and subcutaneous routes, mice were challenged with NMI intraperitoneally. Live NMII vaccination was more protective than that of PhII WCV, demonstrating disease severity similar to that of PhI WCV-immunized mice. These findings highlight the nuances of vaccine composition and potential mechanisms of live bacterial infection that contribute to protective immunity. Despite promising findings, the recent reclassification of NMII RSA439 clone 4 with *cbu0533* mutation reversion calls the feasibility of a live NMII vaccine into question.

Beyond investigating the role of LPS in WCV-induced immunity, additional vaccine formulations have been tested for efficacy in murine models. Dold et al. designed an adenoviral-vectored Q fever vaccine encoding *C. burnetii* protein antigens with 14 surface-expressed protein-encoding genes included in various vaccine formulations (62). A modified hot-phenol LPS extraction method was utilized to isolate *C. burnetii* LPS; however, neither confirmation of LPS length, *C. burnetii* strain identity, nor the LPS quantification method was reported. The addition of *C. burnetii* LPS extract to some adenoviral vector constructs was reported to significantly reduce weight loss following aerosol *C. burnetii* challenge, in contrast to non-LPS-supplemented constructs. Furthermore, LPS-only immunization resulted in less weight loss compared with the empty adenoviral vector group. The authors conclude that although LPS did not affect *C. burnetii* protein-specific immunogenicity, the LPS-induced immune response had the most significant impact on disease reduction (62). While advancements reported in murine study models are promising, they warrant future study of specific mechanisms and the use of more relevant human disease models.

The guinea pig is considered the most physiologically relevant small-animal model of human Q fever (87); however, limited reagent availability hampers the utility of this model. Jan et al. investigated the immunogenicity and protective efficacy of multivalent subunit vaccines supplemented with *C. burnetii* PhI LPS (88). The authors reported that the addition of PhI LPS to an adjuvanted vaccine expressing CBU1910 resulted in dampened immunogenicity via reduction of IgG and IFN-γ production in guinea pig plasma and splenocytes, respectively; however, it is important to note that respective assays were single antigen specific and represent a single time point post immunization and boost. LPS-specific IgG was only detected in guinea pigs administered Q-Vax, the Australian human Q fever vaccine. Following intramuscular immunization and boost, guinea pigs were intratracheally challenged with *C. burnetii* NMI; monovalent and multivalent vaccines appeared to be more protective when supplemented with PhI LPS, based on reduced body temperature and spleen weight following challenge, emphasizing the importance of PhI LPS in protective immunity. In this study, LPS isolation methods were not reported, and LPS characterization (e.g., silver stain, Western blot) was not mentioned. Graves et al. introduced a vaccine comprised of purified *C. burnetii* LPS conjugated to tetanus toxoid; O-antigen, named O-specific polysaccharide (OSP), was procured following a chemical isolation protocol, and lipid A removal was verified by gas chromatography-mass spectrometry (63). In a guinea pig vaccine (intramuscular or subcutaneous)-challenge (intranasal) study, the OSP-toxoid vaccine demonstrated significant protective efficacy. Together, these studies demonstrate the importance of PhI LPS in Q fever vaccine efficacy. Despite this, LPS isolation processes can be onerous and require BSL-3 containment, thereby complicating vaccine development. Long et al. proposed a modified WCV strategy in 2021, utilizing genetically modified PhI NMI and Dugway strains that lack the *dot/icm* secretion system (89). Here, the T4BSS was defined as a *C. burnetii* virulence factor in the guinea pig, the first demonstration in an immunocompetent animal model. As the T4BSS is required for intracellular growth, use of these strains may eventually be recognized as safe in BSL-2 containment (63). Further insight into the role of LPS in vaccine-induced protective immunity and the post-vaccination hypersensitivity (PVH) response was provided by Gregory et al., in 2019, where soluble protein extracts from PhI and PhII *C. burnetii* (61). The authors reported optimal protection against NMI challenge following NMI soluble extract immunization compared with PhII supplemented with PhI LPS. Again, these findings are influenced by the inability to confirm soluble protein and LPS purity, leading the authors to suggest that glycan-protein complexes may be present in the NMI soluble extract, impacting the immune response. Fratzke et al. demonstrated protection against guinea pig aerosol infection challenge utilizing *C. burnetii* (90). The triadjuvant formulation targeting toll-like receptors (TLRs) 4, 7, and 9 prompted the most robust protective efficacy, inducing a Th1 cellular response known to be important for *C. burnetii* protective responses. This study also reported PVH reactogenicity with TLR-adjuvanted subunit formulations containing no LPS, prompting the need for continued study of PVH mechanisms and the specific role of LPS in this response.

Beyond vaccine development, Robison et al. utilized humanized TLR4 and MD-2 adaptor protein (hTLR4/MD-2) mice to investigate the susceptibility of these mice to *C. burnetii* infection (91). Human TLR4 differs in responsiveness to hypo-acylated LPS compared with that of mice. Given the tetra-acylated status of *C. burnetii* LPS, it was logical that intratracheal infection in hTLR4-MD2 mice resulted in enhanced lung bacterial burdens and lung pathology compared with wild-type mice. Bone marrow chimera experiments with wild-type and hTLR4-MD-2 mice demonstrated enhanced splenic bacterial burden but only with stromal/epithelial cell hTLR4/MD-2 expression. The timing of these effects was distinct and suggested an early role for hTLR4/MD-2 in immune cell recruitment and function, leading to enhanced disease.

### LPS methodology

In 2023, Anderson et al. reported a simple method of PhI LPS-expressing bacterial isolation (92). This method involved passaging mixed-phase *C. burnetii* cultures on Vero cells. Likely due to the autoagglutination properties of PhII LPS, purified PhI LPS cultures were collected after overnight incubation, as demonstrated by silver stain and Western blot. This method offers an animal-free alternative to *in vivo* passage for obtaining PhI LPS-expressing bacteria. Though similar sugar derivatization was employed, Webb et al. used liquid chromatograph mass spectrometry (LCMS) to provide more sensitive detection methods for virenose and dihydrohydroxystreptose in *C. burnetii* LPS monosaccharide composition. This method was proposed as a detection assay for PhI-expressing bacteria (93).

## COMMENTARY

### Biosafety implications

Since the pathogen was identified and propagated in laboratories, *C. burnetii* has been a leading cause of laboratory-acquired infections (LAIs) (94). Contributing factors include the organism’s low infectious dose, aerosol transmission route, and environmental stress tolerance. Early (1930–1970) and more recent (1979–2015) systematic reviews identify *C. burnetii* as the cause of 12.9% and 11.6% of LAIs, respectively. Thus, *C. burnetii* is classified as a select agent by the US FSAP, a BSL-3 organism by the European Center for Disease Prevention and Control (95), and a Risk Group 3 organism by the NIH Guidelines (96). The most current edition of the US CDC Biosafety in Microbiological and Biomedical Laboratories advises biosafety level 3 (BSL-3) and animal biosafety level 3 (ABSL-3) containment for most work involving *C. burnetii*, except for non-propagative diagnostic procedures (97). Since the early 2000s, the NMII, clone 4 strain has been excluded from these classifications and was designated as safe to handle in a BSL-2 setting (67). The basis of NMII, clone 4 FSAP exclusion included the large genetic deletion described by Hoover et al., thought to be responsible for PhII LPS paired with the lack of disease and bacterial recovery in a guinea pig infection model (64, 98). All of these concepts have been challenged by the recent description of LPS elongation by NMII, clone 4, driven by a 3-bp, seemingly promiscuous genetic lesion that can confer enhanced virulence in a guinea pig infection model (74). Indeed, NMII strains with elongated LPS can exhibit virulence comparable to that of NMC (74), which is classified as an FSAP select agent and requires BSL-3 containment. The US FSAP states that “An excluded select agent strain or modified toxin will be subject to the regulations if there is a reintroduction of factor(s) associated with virulence, toxic activity, or other manipulations that modify the attenuation such that virulence or toxic activity is restored or enhanced” (81). The FSAP NMII RSA439 clone 4 exclusion description now includes the following guidance: “*Coxiella burnetii* Phase II, Nine Mile Strain, plaque purified clone 4 with reversion to wild-type *cbu0533* is a select agent and subject to select agent regulations. This revertant strain displayed an intermediate length LPS phenotype under certain growth conditions and could cause infection in guinea pigs and could be isolated from their spleens” (81). Thus, if NMII RSA439 clone 4, which is commonly manipulated as a non-select agent in BSL-2 containment, exhibits *cbu0533* mutation reversion, this guidance necessitates submission of a USDA/CDC Form 4 to the Federal Select Agent Program, reporting identification of a select agent. This potential scenario extends to laboratory manipulation of NMII RSA439 clone 4, even after *cbu0533* mutational screening, as reversion could theoretically happen at any time. Currently, intraperitoneal guinea pig infection at doses > 10^6^ and passaging in ACCM-1 have resulted in NMII *cbu0533* mutation reversion and LPS elongation; however, ongoing studies will likely reveal additional conditions that foster mutation reversion.

Recently, Blacksell et al. presented important gaps in scientific evidence and practice regarding *C. burnetii* and biosafety, including limitations in LAI reporting, disinfection efficacy, and one health integration, highlighting the importance of appropriate risk assessment and biosafety-related precautions for safe laboratory manipulation of this pathogen (99). There is currently no evidence of LAI from *C. burnetii* NMII with elongated LPS; however, Q fever can be asymptomatic or present with non-specific, flu-like symptoms (19, 20), complicating surveillance. Given the enhanced virulence observed in guinea pigs following *cbu0533* mutation reversion and LPS elongation, the FSAP’s decision reflects the elevated risk associated with NMII LPS elongation. Further risk analysis for the NMII RSA439 clone 4 with *cbu0533* mutation reversion may be warranted; however, this falls within the purview of biosafety professionals. To allay some of these concerns, the NMII RSA439 clone 4 Δ*cbu0533* strain has been deposited with BEI Resources and is available at no cost to eligible, registered researchers (100). We encourage researchers to work closely with their biosafety colleagues to perform a detailed risk assessment and to ensure compliance with applicable institutional and federal policies if work with this strain at BSL2 is requested.

### Remaining knowledge gaps

Since 2017, advancements have been made in the study of *C. burnetii* LPS structure, genetics, and function through novel experimental approaches and exploration of unanswered hypotheses; however, major questions remain and warrant future research. Many of these knowledge gaps relate to LPS structure, including a complete structural understanding of *C. burnetii* lipid A and O-antigen and the nature of LPS structural heterogeneity among *C. burnetii* strains. Several studies focusing on *C. burnetii* LPS structure and bioactivity use *E. coli* or *Salmonella* spp. as “controls,” despite the expansive heterogenicity of LPS structure among bacteria of even the same species. More exhaustive comparisons of LPS structure and bioactivity with bacteria more closely related to *C. burnetii* could also be informative and provide insight on the evolutionary changes to LPS structure over time. Tertra-acylated lipid A is particularly interesting, as it is heavily believed to be an evolutionary benefit resulting in decreasing TLR signaling. Other bacteria such as *Yersinia pestis* and *Francisella tularensis* have hypo-acylated lipid A structures (101, 102). Genetic knowledge gaps include an incomplete understanding of genes responsible for lipid A biosynthesis, O-antigen biosynthesis, and phase variation. The assessment of growth conditions under which LPS elongation can occur is incomplete and warrants further study, both experimentally and in respect to biosafety. In the context of LPS and host immunity, the mechanisms underlying LPS phase and the development of protective immunity are not fully elucidated, and related studies utilizing biologically relevant models of human disease would help to close this critical knowledge gap, informing Q fever vaccine design.

Clearly, LPS phase can have wide-ranging impacts on many areas of *C. burnetii* biology, including host-pathogen interactions and pathogenesis. The lack of comprehensive LPS characterization in experiments in which LPS length may affect results is of concern, particularly in the context of NMII LPS elongation. Similarly, methods used for LPS extraction and isolation are often described in insufficient detail to ensure reproducibility. Ideally, inclusion of LPS characterization and methodological data will be more commonplace in *C. burnetii* studies. Similarly, the application of experimental methods, such as expansion of LPS-recognizing antibodies and advanced LPS chemical analysis, would expand our understanding of *C. burnetii* LPS structure and function. *C. burnetii* LPS work is restricted to *in vitro* studies, given that the bacteria isolated from an infected animal are typically grown out *in vitro* for LPS analysis. Work with *K. pneumoniae* and *Pseudomonas aeruginosa* has demonstrated that lipid A structures can vary *in vivo* compared with *in vitro* experiments (53, 103, 104). This is an important consideration when drawing conclusions about the structure of *C. burnetii* LPS.

**Fig 3:**
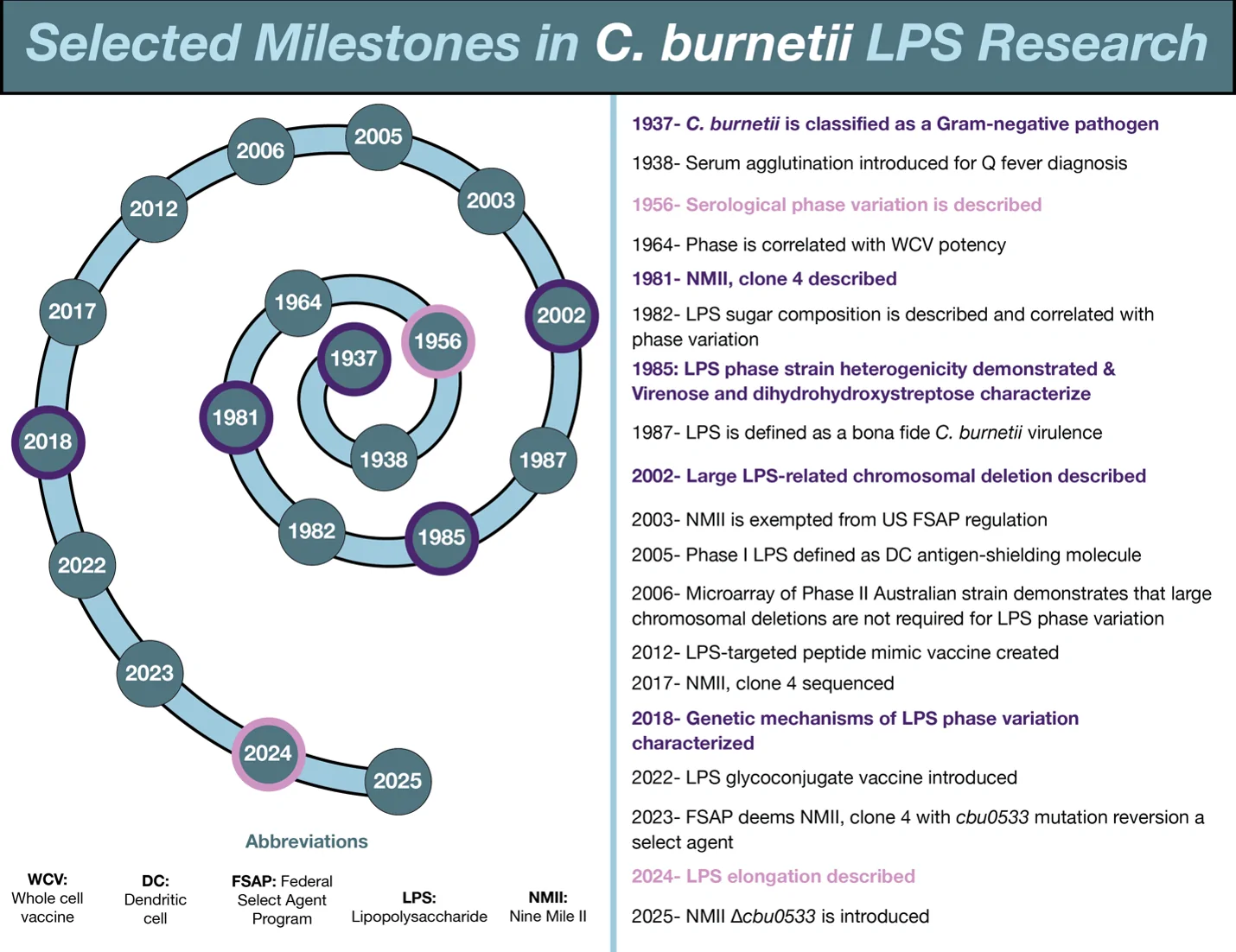
Selected milestones in *C. burnetii* LPS research.

By expanding our understanding of *C. burnetii* LPS, the development of Q fever countermeasures will be enhanced. Further, insights may be related to other intracellular pathogens for which countermeasure development is challenging. Historical milestones in *C. burnetii* LPS research have been complemented by recent studies, leading to a more complete understanding of the structure and function of this fascinating molecule (Fig. 3). Given the ubiquitous functional impact of *C. burnetii* LPS on disease, without seminal advances such as the identification of serological phase variation in 1956, the correlation with LPS length in 1982, and the identification of LPS-related genetic lesions in 2002, the study of *C. burnetii* would be impaired. As predicted by Hoover et al., in 2003, LPS-related genetic lesions outside of the deletions identified the year prior would later be proven in recent years (65, 68). The identification of *cbu0533* as a gene related to LPS biosynthesis and phase variation in 2018 led to the recent identification of LPS elongation, raising newfound regulatory and biosafety concerns regarding experimental manipulation of the laboratory workhorse NMII RSA439 clone 4 strain. The study of *C. burnetii* LPS will continue to inform advances in fundamental understanding of this bacterial pathogen and may have broader implications for bacteriology, immunology, and vaccinology. When it comes to *C. burnetii* LPS, what goes around comes around, and the future is as intriguing as the past.
